# U.S. Adolescent Rest-Activity patterns: insights from functional principal component analysis (NHANES 2011–2014)

**DOI:** 10.1186/s12966-023-01520-3

**Published:** 2023-10-13

**Authors:** Chris Ho Ching Yeung, Jiachen Lu, Erica G. Soltero, Cici Bauer, Qian Xiao

**Affiliations:** 1 Department of Epidemiology, Human Genetics and Environmental Sciences, School of Public Health, University of Texas Health Science Center at Houston, 1200 Pressler St., Houston, TX USA; 2 Department of Biostatistics and Data Science, School of Public Health, School of Public Health, University of Texas Health Science Center at Houston, Houston, TX USA; 3 United States Department of Agriculture/Agricultural Research Services Children’s Nutrition Research Center, Department of Pediatrics, Baylor College of Medicine, Houston, TX 77030 USA; 4 Center for Spatial‑Temporal Modeling for Applications in Population Sciences, School of Public Health, University of Texas Health Science Center at Houston, Houston, TX USA

**Keywords:** Rest-activity rhythm, Circadian rhythm, Functional principal component analysis, Adolescence, NHANES, Accelerometer

## Abstract

**Background:**

Suboptimal rest-activity patterns in adolescence are associated with worse health outcomes in adulthood. Understanding sociodemographic factors associated with rest-activity rhythms may help identify subgroups who may benefit from interventions. This study aimed to investigate the association of rest-activity rhythm with demographic and socioeconomic characteristics in adolescents.

**Methods:**

Using cross-sectional data from the nationally representative National Health and Nutrition Examination Survey (NHANES) 2011–2014 adolescents (N = 1814), this study derived rest-activity profiles from 7-day 24-hour accelerometer data using functional principal component analysis. Multiple linear regression was used to assess the association between participant characteristics and rest-activity profiles. Weekday and weekend specific analyses were performed in addition to the overall analysis.

**Results:**

Four rest-activity rhythm profiles were identified, which explained a total of 82.7% of variance in the study sample, including (1) High amplitude profile; (2) Early activity window profile; (3) Early activity peak profile; and (4) Prolonged activity/reduced rest window profile. The rest-activity profiles were associated with subgroups of age, sex, race/ethnicity, and household income. On average, older age was associated with a lower value for the high amplitude and early activity window profiles, but a higher value for the early activity peak and prolonged activity/reduced rest window profiles. Compared to boys, girls had a higher value for the prolonged activity/reduced rest window profiles. When compared to Non-Hispanic White adolescents, Asian showed a lower value for the high amplitude profile, Mexican American group showed a higher value for the early activity window profile, and the Non-Hispanic Black group showed a higher value for the prolonged activity/reduced rest window profiles. Adolescents reported the lowest household income had the lowest average value for the early activity window profile.

**Conclusions:**

This study characterized main rest-activity profiles among the US adolescents, and demonstrated that demographic and socioeconomic status factors may shape rest-activity behaviors in this population.

**Supplementary Information:**

The online version contains supplementary material available at 10.1186/s12966-023-01520-3.

## Background

Human rest-activity rhythms typically follow a 24-hour cycle that are regulated by the internal circadian clock and entrained by external factors such as light exposure and social interactions [1]. Using accelerometer data, previous studies derived parametric and nonparametric metrics to capture features of 24-hour rest-activity rhythms [2–4]. Although these commonly-used methods can generate useful quantifiers of rest-activity rhythms, they either rely on specific model assumptions or apply a set of fixed algorithms and thus lack flexibility for characterizing real-life rest-activity rhythms in diverse samples. Alternatively, functional principal component analysis (fPCA) represents a flexible approach to fit time series data with minimal assumptions about the assumed shape of 24-hour rest-activity patterns. By fitting individual data with Fourier-based functions, the fPCA extracts activity profiles that explain the largest percentages of variance in rest-activity patterns in the population. Previous studies have applied fPCA in diverse human populations to derive multiple profiles of rest-activity rhythms, some of which (e.g., biphasic pattern) representing novel features that were not previously revealed by conventional methods [5]. Moreover, some of these fPCA profiles have been linked with health and disease outcomes, including Alzheimer’s disease [6], mood states [7], depressive and anxiety disorders [8], cognition and mortality [9].

Adolescence is a critical period in human development and is often associated with changes in rest-activity patterns [10–12]. Studies of the U.S. adolescents have shown an increase in sedentary behavior, and a decline in physical activity levels and sleep duration in the last two to three decades [13–16], and these trends accompanied rising prevalence of obesity and metabolic syndrome in this population [17–19]. Suboptimal rest-activity patterns have been shown to be associated with adverse health outcomes in adolescents, including obesity and lower cardiorespiratory fitness, mental health problems, and poor academic performance [20–23]. Previous studies have also shown that physical inactivity and sleep deficiency during adolescence were significant predictors of health outcomes in adulthood [24–26], highlighting the importance of early life exposures. Like adults, rest-activity rhythms in adolescents are likely shaped by both biological and socioenvironmental factors. Given the myriad of health implications that are associated with rest and activity behaviors in the adolescents, and the worsening rest-activity patterns in the U.S. adolescents, it is important for public health research to identify sociodemographic correlates of rest-activity rhythms to identify vulnerable subgroups that may benefit from interventions aiming at improving sleep, physical activity and overall rhythmic patterns. Therefore, the objective of this study is to apply fPCA to derive rest-activity profiles from accelerometer data, and study their association with demographic and socioeconomic characteristics in a nationally representative sample of U.S. adolescents.

## Methods

### Study population

The National Health and Nutrition Examination Survey (NHANES) is an biennial survey that enrolls a nationally representative sample of the civilian noninstitutionalized population to assess the health and nutritional status of the U.S. adults and children through interviews, medical examination and laboratory tests [27]. The NHANES study was reviewed and approved by the National Center for Health Statistics Research Ethics Review Board [28]. The current study based on data from the 2011–2014 cycle in which 24-hour accelerometer recordings were collected. Among the 19,931 participants, 2705 were 12–19 years old. Of these, we excluded 891 participants with less than four days of valid accelerometer data (a valid day was defined as having 20 h or more of usable wake or sleep data according to previously reported criteria [29]). The final analytic sample included 1814 participants. Table S1 compared demographic characteristics among participants (aged 12 to 19) by inclusion status. The two groups were largely similar with regard to most of the sociodemographic characteristics with the exception of age: the excluded participants were generally older when compared to those included in the analytic sample.

### Rest-activity profiles

Accelerometer movement data were collected from all participants aged three years or older by ActiGraph model GT3X + which measures triaxial acceleration every 1/80 of a second [30]. Participants were asked to wear the physical activity monitor on the non-dominant wrist for seven consecutive days [29]. The quality of data was reviewed and each 1-minute summary epoch was used to classify the epoch as wake, sleep, non-wear or unknown using a published algorithm [29].

fPCA was performed to derive rest-activity profiles using the summary triaxial acceleration measure, Monitor Independent Movement Summary (MIMS) [31]. Details about this open-source summary metric have been reported before [31]. Briefly, it is a device-independent measure optimized for capturing human movements and demonstrated higher consistency across devices than other summary metrics as well as increased sensitivity in detecting wrist movements during sedentary periods. For each participant, we first summarized the MIMS value into 5-minute epochs, and then calculated the mean value for each 5-minute epoch across all days. The use of 5-minute epochs helped preserving temporal information for identifying population-level patterns while minimizing noise and reducing data missingness. We applied Fourier basis functions to decompose the mean activity data and fit a functional curve that represents a smoothed activity profile. Fourier basis is chosen for data sampled in fixed frequency [32]. Functional principal component (fPC) scores were returned after performing fPCA on the smoothed activity profiles from all participants. Each fPC score was standardized by sample mean and variance. In this analysis we focused on the top four components that collectively explained more than 80% variability in the data using established criteria [5–9].

### Participants characteristics

Demographic and socioeconomic information was collected from participants and their family by trained interviewers. We included the following in this study: 1) age [12–19], sex (boys, girls), race/ethnicity (non-Hispanic White, non-Hispanic Black, Mexican American, Other Hispanic, Asian, and others), and household income (<$20k, $20k-$44.9k, $45k-$74.9k, ≥$75k). We also included the higher level of education reported by the household reference person for themselves and their spouse (less than 9th grade, 9-11th grade, high school graduate, some college, college graduate or higher) as the measure of parental education.

### Statistical analysis

Multiple linear regression was used to assess the association between participant characteristics and rest-activity profiles. Participant characteristics were treated as the explanatory, and the fPC values were treated as the outcome variables. For categorical variables, we chose the group presumed as having the most healthy rest-activity pattern as the reference, i.e., the youngest group [12, 13] for age, boys for sex, Non-Hispanic White for race/ethnicity, ≥$75k for household income, and college graduate or higher for parental education [5]. As the activity patterns are likely to differ between the weekdays and weekends, we performed weekday- and weekend-specific analyses in addition to the overall analysis. To account for the complex sample design, NHANES full sample mobile examination center exam weight was applied in the regression analysis. All analyses were performed in R, with R package fda (v6.0.5) for the fPCA analysis.

## Results

**Fig. 1:**
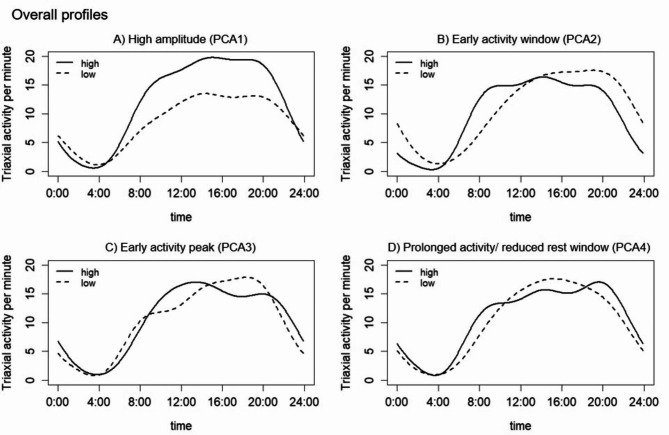
Rest-activity profiles of 24-hour accelerometer data from adolescents in the National Health and Nutrition Examination Survey (2011–2014). Each panel depicts the mean 24-hour activity patterns for participants with high (solid line) and low (dotted) eigenvalues of the first four components derived from the functional principal component analysis (PCA): (**A**) The first component (42.8% variance), with higher eigenvalues representing a higher amplitude; (**B**) the second component (24.4% variance), with higher eigenvalues representing earlier activity window; (**C**) the third component (8.3% variance), with higher eigenvalues representing earlier peak of daytime activity; (**D**) the forth component (7.1% variance), with higher eigenvalues representing prolonged activity/reduced rest window

**Fig. 2:**
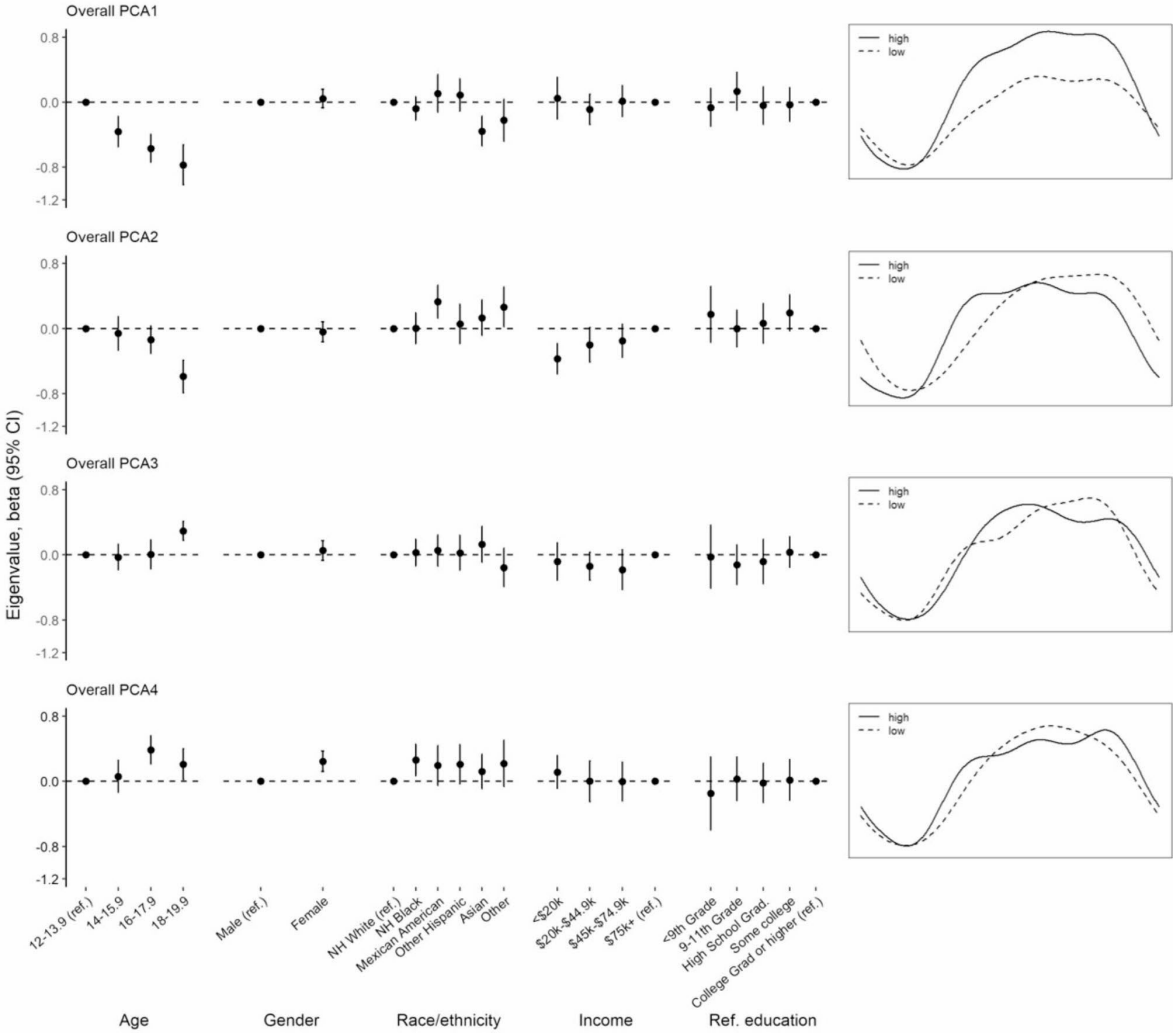
Associations between participant characteristics and overall rest-activity profiles in adolescence in the National Health and Nutrition Examination Survey (2011–2014). Multiple linear regression models included all participant characteristics simultaneously. Abbreviations: CI, confidence interval; HS, high school; NH, non-Hispanic; PCA1-4, principal component analysis component 1

Figure 1 shows the rest-activity profiles of the top four PCA components which explained a total of 82.7% of variance in the accelerometer movement data. The four PCA components are: (1) High amplitude profile (PCA1), where a higher value reflected a higher day-time physical activity level (Fig. 1A); (2) Early activity window profile (PCA2), where a higher value reflected an earlier and steeper increase in activity level in the morning, accompanied by an earlier decline of activity in the evening (Fig. 1B); (3) Early activity peak profile (PCA3), where a higher value reflected an earlier timing of the peak of daily activity (Fig. 1 C); and (4) Prolonged activity/reduced rest window profile (PCA4), where a higher value reflected a longer active period or shorter rest period (Fig. 1D). The weekday- and weekend-specific profiles are shown in Figure S1 & S2. The rest-activity profiles for weekdays were similar to and highly correlated (correlation coefficients (r): 0.88–0.95) with the overall profiles (Table S2 and Figure S3). The PCA1 and PCA2 for weekends were also largely similar to that of the overall profiles (r: 0.80 for PCA1 and 0.70 for PCA2). However, the PCA3 and PCA4 of weekend profiles seemed to correspond to the PCA4 and PCA3 for overall profiles, suggesting a switch in their relative importance in explaining total sample variabilities. Notably, the weekend PCA3 showed a biphasic pattern, instead of the three-peak pattern observed for overall and weekday PCA4, suggesting that the latter may correspond to the more active, lunch-time recess window that is absent on weekends.

Associations between participant characteristics and overall rest-activity profiles are shown in Fig. 2; Table 1. Age was associated with all four rest-activity profiles. When compared to the youngest group (12-13.9), older age groups showed a lower value for the high amplitude (PCA1) and early activity window (PCA2) profiles, but a higher value for the early activity peak (PCA3) and prolonged activity/reduced rest window (PCA4) profiles. Sex was only associated with the prolonged activity/reduced rest window profile (PCA4), with girls showing a higher eigenvalue. When compared to Non-Hispanic White adolescents, Asian showed a lower value for the high amplitude profile (PCA1), Mexican American group showed a higher value for the early activity window profile (PCA2), and the Non-Hispanic Black group on average showed significantly higher values for the prolonged activity/reduced rest window (PCA4) profile. Compared to the adolescents with the highest household income, the lowest household income group had a lower value for the early activity window profile (PCA2). No association was found with parental education.

**Table 1:** Associations between participant characteristics and overall rest-activity profiles in adolescents in the National Health and Nutrition Examination Survey (2011–2014)

	Overall PCA1		Overall PCA2		Overall PCA3		Overall PCA4	
	Median (IQR)	Beta (95% CI)	Median (IQR)	Beta (95% CI)	Median (IQR)	Beta (95% CI)	Median (IQR)	Beta (95% CI)
**Age**								
12-13.9	0.48 (-0.08, 1.04)	Ref.	0.31 (-0.33, 0.77)	Ref.	-0.06 (-0.75, 0.51)	Ref.	-0.20 (-0.86, 0.44)	Ref.
14-15.9	0.09 (-0.55, 0.63)	-0.36 (-0.55, -0.17)	0.28 (-0.34, 0.86)	-0.06 (-0.26, 0.14)	-0.12 (-0.61, 0.62)	-0.03 (-0.18, 0.13)	-0.24 (-0.80, 0.54)	0.06 (-0.14, 0.25)
16-17.9	-0.18 (-0.69, 0.52)	-0.57 (-0.74, -0.40)	0.13 (-0.45, 0.71)	-0.14 (-0.31, 0.03)	-0.06 (-0.61, 0.50)	0.01 (-0.17, 0.19)	0.16 (-0.45, 0.87)	0.39 (0.21, 0.56)
18-19.9	-0.42 (-1.02, 0.34)	-0.77 (-1.02, -0.53)	-0.34 (-1.02, 0.26)	-0.59 (-0.79, -0.39)	0.21 (-0.44, 0.75)	0.29 (0.18, 0.41)	-0.10 (-0.71, 0.46)	0.21 (0.02, 0.40)
**Sex**								
Male	-0.01 (-0.73, 0.70)	Ref.	0.21 (-0.55, 0.70)	Ref.	-0.06 (-0.67, 0.66)	Ref.	-0.22 (-0.79, 0.40)	Ref.
Female	0.08 (-0.55, 0.71)	0.05 (-0.07, 0.16)	0.09 (-0.61, 0.67)	-0.04 (-0.16, 0.08)	0.01 (-0.51, 0.53)	0.05 (-0.07, 0.18)	0.07 (-0.62, 0.67)	0.25 (0.12, 0.37)
**Race/ethnicity**								
NH Black	-0.05 (-0.70, 0.65)	-0.08 (-0.22, 0.06)	0.06 (-0.81, 0.62)	0.00 (-0.19, 0.19)	0.00 (-0.62, 0.68)	0.03 (-0.13, 0.19)	0.12 (-0.53, 0.70)	0.26 (0.07, 0.46)
Mexican American	0.17 (-0.46, 0.75)	0.11 (-0.12, 0.34)	0.36 (-0.28, 0.86)	0.33 (0.13, 0.53)	-0.11 (-0.64, 0.54)	0.05 (-0.14, 0.25)	-0.03 (-0.75, 0.60)	0.19 (-0.05, 0.44)
Other Hispanic	0.15 (-0.44, 0.76)	0.09 (-0.11, 0.29)	0.05 (-0.61, 0.63)	0.06 (-0.19, 0.30)	0.01 (-0.62, 0.61)	0.03 (-0.19, 0.24)	-0.12 (-0.63, 0.57)	0.21 (-0.03, 0.45)
Asian	-0.32 (-0.92, 0.31)	-0.35 (-0.54, -0.17)	0.19 (-0.56, 0.73)	0.13 (-0.09, 0.35)	0.06 (-0.44, 0.61)	0.13 (-0.09, 0.35)	-0.05 (-0.57, 0.44)	0.12 (-0.09, 0.33)
Other	-0.04 (-0.87, 0.43)	-0.22 (-0.48, 0.03)	0.29 (-0.22, 0.82)	0.27 (0.02, 0.51)	-0.35 (-0.77, 0.38)	-0.16 (-0.39, 0.08)	-0.10 (-0.55, 0.63)	0.22 (-0.07, 0.51)
NH White	0.05 (-0.59, 0.71)	Ref.	0.11 (-0.62, 0.63)	Ref.	0.00 (-0.57, 0.57)	Ref.	-0.17 (-0.83, 0.46)	Ref.
**Household income**								
<$20k	0.00 (-0.63, 0.69)	0.05 (-0.21, 0.30)	-0.12 (-0.84, 0.51)	-0.37 (-0.56, -0.19)	0.02 (-0.63, 0.73)	-0.08 (-0.31, 0.15)	0.02 (-0.54, 0.63)	0.11 (-0.09, 0.32)
$20k-$44.9k	0.03 (-0.70, 0.71)	-0.09 (-0.27, 0.10)	0.16 (-0.60, 0.74)	-0.2 (-0.41, 0.01)	-0.06 (-0.67, 0.49)	-0.14 (-0.31, 0.03)	-0.04 (-0.71, 0.65)	-0.00 (-0.25, 0.25)
$45k-$74.9k	0.11 (-0.53, 0.66)	0.01 (-0.18, 0.20)	0.17 (-0.47, 0.64)	-0.15 (-0.36, 0.05)	-0.12 (-0.64, 0.49)	-0.18 (-0.43, 0.07)	-0.13 (-0.66, 0.48)	-0.00 (-0.24, 0.24)
$75k+	0.04 (-0.59, 0.73)	Ref.	0.22 (-0.45, 0.73)	Ref.	0.03 (-0.50, 0.67)	Ref.	-0.13 (-0.76, 0.46)	Ref.
**HH ref person’s education level**								
Less Than 9th Grade	0.18 (-0.58, 0.71)	-0.06 (-0.30, 0.17)	0.31 (-0.55, 0.90)	0.17 (-0.17, 0.51)	0.00 (-0.51, 0.60)	-0.02 (-0.41, 0.36)	-0.20 (-0.98, 0.54)	-0.15 (-0.6, 0.30)
9-11th Grade	-0.01 (-0.64, 0.88)	0.13 (-0.10, 0.36)	-0.03 (-0.74, 0.45)	-0.00 (-0.23, 0.22)	-0.14 (-0.70, 0.53)	-0.12 (-0.36, 0.12)	0.02 (-0.58, 0.66)	0.03 (-0.24, 0.29)
High School Grad	0.07 (-0.72, 0.66)	-0.04 (-0.27, 0.19)	0.13 (-0.49, 0.64)	0.06 (-0.18, 0.31)	-0.06 (-0.71, 0.45)	-0.08 (-0.35, 0.19)	-0.05 (-0.76, 0.56)	-0.02 (-0.26, 0.22)
Some College	-0.05 (-0.69, 0.52)	-0.03 (-0.23, 0.18)	0.20 (-0.55, 0.75)	0.19 (-0.03, 0.42)	0.01 (-0.63, 0.66)	0.03 (-0.15, 0.22)	-0.10 (-0.68, 0.51)	0.01 (-0.23, 0.26)
College Graduate	0.12 (-0.51, 0.76)	Ref.	0.15 (-0.53, 0.64)	Ref.	0.00 (-0.54, 0.61)	Ref.	-0.11 (-0.68, 0.56)	Ref.

Abbreviations: CI confidence interval, HH Household, IQR interquartile range, NH non-Hispanic, PCA1-4 principal component analysis component 1-4

Beta (95% CI) derived from multiple linear regression models included all study characteristics and their categories simultaneously

Figure S4, S5 and Table 2 presented the associations between participant characteristics and weekday and weekend rest-activity profiles. The associations found for the weekday profiles were largely similar to those for the overall profiles. For the weekend profiles, results for the high amplitude (PCA1) and the early activity window (PCA2) profiles were also similar to those for the overall profiles, albeit with two notable exceptions: First, girls showed a significantly greater value for the high amplitude (PCA1) weekend profile compared with boys, which was not observed in overall or weekday analysis. Second, the relationship between the Mexican American group and the early activity window profile (PCA2) for weekday and overall analysis was not observed for weekend profiles. Finally, because the PCA3 and PCA4 weekend profiles largely resembled the PCA4 and PCA3 profiles in overall analysis, the associations with these rest-activity profiles also appeared broadly similar. However, the relationship between non-Hispanic Black group and the prolonged activity/reduced rest window profile was attenuated for the weekend profile (weekend PCA3).

**Table 2:** Associations (beta (95%CI)) between participant characteristics and rest-activity profiles for weekday and weekend in adolescents in the National Health and Nutrition Examination Survey (2011–2014)

	PCA1		PCA2		PCA3		PCA4	
	Weekday	Weekend	Weekday	Weekend	Weekday	Weekend	Weekday	Weekend
**Age**								
12-13.9	Ref.	Ref.	Ref.	Ref.	Ref.	Ref.	Ref.	Ref.
14-15.9	-0.32 (-0.51, -0.14)	-0.32 (-0.49, -0.15)	0.02 (-0.17, 0.21)	-0.11 (-0.32, 0.09)	-0.14 (-0.32, 0.04)	0.1 (-0.06, 0.25)	-0.01 (-0.19, 0.17)	0.37 (0.14, 0.60)
16-17.9	-0.56 (-0.74, -0.38)	-0.46 (-0.64, -0.29)	-0.07 (-0.23, 0.09)	-0.07 (-0.29, 0.15)	-0.23 (-0.38, -0.08)	0.55 (0.35, 0.76)	0.19 (0.03, 0.35)	0.16 (-0.08, 0.39)
18-19.9	-0.87 (-1.11, -0.62)	-0.55 (-0.77, -0.32)	-0.48 (-0.65, -0.3)	-0.39 (-0.59, -0.18)	0.17 (0.02, 0.32)	0.55 (0.38, 0.72)	0.07 (-0.07, 0.21)	0.30 (0.10, 0.51)
**Sex**								
Male	Ref.	Ref.	Ref.	Ref.	Ref.	Ref.	Ref.	Ref.
Female	-0.00 (-0.12, 0.11)	0.15 (0.01, 0.29)	-0.01 (-0.12, 0.10)	0.05 (-0.11, 0.20)	-0.03 (-0.17, 0.11)	0.19 (0.02, 0.36)	0.27 (0.14, 0.40)	-0.07 (-0.17, 0.03)
**Race/ethnicity**								
NH Black	-0.03 (-0.18, 0.12)	-0.11 (-0.24, 0.02)	0.06 (-0.12, 0.24)	-0.1 (-0.29, 0.09)	-0.04 (-0.22, 0.14)	0.15 (-0.02, 0.32)	0.19 (-0.01, 0.39)	-0.01 (-0.19, 0.18)
Mexican American	0.20 (-0.05, 0.45)	-0.06 (-0.27, 0.15)	0.32 (0.12, 0.51)	0.17 (-0.04, 0.37)	0.04 (-0.19, 0.26)	-0.02 (-0.21, 0.18)	0.12 (-0.11, 0.36)	-0.08 (-0.28, 0.13)
Other Hispanic	0.15 (-0.05, 0.35)	-0.04 (-0.25, 0.17)	0.07 (-0.17, 0.31)	-0.07 (-0.32, 0.17)	0.01 (-0.25, 0.27)	0.04 (-0.14, 0.23)	0.17 (-0.06, 0.40)	-0.10 (-0.30, 0.10)
Asian	-0.27 (-0.45, -0.08)	-0.43 (-0.61, -0.24)	0.21 (-0.02, 0.44)	-0.01 (-0.22, 0.21)	0.10 (-0.12, 0.33)	0.05 (-0.11, 0.20)	0.11 (-0.08, 0.30)	-0.09 (-0.28, 0.11)
Other	-0.16 (-0.48, 0.17)	-0.22 (-0.46, 0.02)	0.33 (0.03, 0.63)	0.08 (-0.17, 0.33)	-0.12 (-0.36, 0.13)	-0.18 (-0.59, 0.23)	0.25 (-0.00, 0.50)	-0.17 (-0.55, 0.21)
NH White	Ref.	Ref.	Ref.	Ref.	Ref.	Ref.	Ref.	Ref.
**Household income**								
<$20k	0.03 (-0.23, 0.28)	0.05 (-0.20, 0.29)	-0.32 (-0.49, -0.15)	-0.36 (-0.58, -0.14)	-0.07 (-0.31, 0.18)	0.09 (-0.11, 0.30)	0.07 (-0.11, 0.26)	-0.05 (-0.33, 0.23)
$20k-$44.9k	-0.06 (-0.23, 0.12)	-0.13 (-0.35, 0.08)	-0.13 (-0.35, 0.09)	-0.25 (-0.50, -0.01)	-0.14 (-0.33, 0.06)	0.06 (-0.17, 0.30)	-0.05 (-0.3, 0.19)	-0.07 (-0.34, 0.20)
$45k-$74.9k	-0.00 (-0.22, 0.21)	-0.01 (-0.21, 0.19)	-0.16 (-0.38, 0.07)	-0.13 (-0.37, 0.11)	-0.24 (-0.48, 0.00)	0.17 (-0.09, 0.42)	-0.10 (-0.34, 0.14)	0.03 (-0.20, 0.26)
≥$75k	Ref.	Ref.	Ref.	Ref.	Ref.	Ref.	Ref.	Ref.
**HH ref person’s education level**								
Less Than 9th Grade	-0.08 (-0.34, 0.18)	0.02 (-0.22, 0.27)	0.19 (-0.11, 0.48)	0.08 (-0.27, 0.43)	-0.00 (-0.39, 0.38)	-0.12 (-0.34, 0.10)	-0.13 (-0.52, 0.26)	-0.07 (-0.43, 0.29)
9-11th Grade	0.15 (-0.07, 0.38)	0.15 (-0.10, 0.40)	0.07 (-0.14, 0.28)	-0.20 (-0.49, 0.08)	-0.12 (-0.39, 0.15)	-0.14 (-0.35, 0.07)	0.05 (-0.20, 0.30)	-0.01 (-0.28, 0.26)
High School Grad	-0.02 (-0.24, 0.20)	0.00 (-0.25, 0.25)	0.14 (-0.09, 0.37)	-0.11 (-0.37, 0.14)	-0.06 (-0.32, 0.21)	-0.23 (-0.45, -0.01)	0.06 (-0.19, 0.31)	-0.07 (-0.29, 0.16)
Some College	0.01 (-0.19, 0.21)	-0.00 (-0.22, 0.22)	0.24 (0.04, 0.45)	0.01 (-0.23, 0.24)	0.02 (-0.21, 0.24)	-0.04 (-0.23, 0.16)	0.00 (-0.23, 0.23)	-0.04 (-0.24, 0.16)
College Graduate or higher	Ref.	Ref.	Ref.	Ref.	Ref.	Ref.	Ref.	Ref.

Abbreviations: CI confidence interval, HH Household, NH non-Hispanic, PCA1-4 principal component analysis component 1-4

Beta (95% CI) derived from multiple linear regression models included all study characteristics and their categories simultaneously

## Discussion

In NHANES 2011–2014, we identified the top four rest-activity profiles of adolescents with the use of fPCA. Both correlations and differences were observed between weekday and weekend profiles. Moreover, we observed notable variation of these rest-activity profiles across subgroups of age, sex, race/ethnicity, and household income, suggesting broad influences from demographic characteristics and family socioeconomic status.

### High amplitude profile

PCA1 was primarily a measure of day-time activity level, and explained the largest portion of variance (42.8%) in rest-activity profiles, similar to previous studies in adults [5, 8]. The high amplitude profile was strongly and inversely associated with age. This is consistent with a large body of literature showing a decline in physical activity with age in adolescents [33, 34]. Physical inactivity is an important risk factors for numerous diseases, such as obesity and diabetes [35]. Moreover, health behaviors established in adolescence have been shown to track into adulthood [36], and may affect health outcomes later in life [24, 25]. Preventing the decline of physical activity in adolescents, for example through multicomponent intervention involving schools, families and communities [37], is an important public health priority. Other than older age, a lower amplitude was also observed in Asian adolescents compared with non-Hispanic whites, which agrees with previous studies showing a lower activity level in Asian adolescents and adults [38, 39].

The weekday and weekend profiles showed a relatively high correlation (0.59) and were similarly associated with demographic and family socioeconomic status. However, although boys and girls were similar with regard to the high amplitude profile on weekdays, girls showed a greater value for this profile on weekends. Given that the high amplitude profile appeared to be primarily driven by day-time activity levels, this finding appeared somewhat unexpected as previous studies generally showed a higher physical activity level among boys than girls [40]. However, most of the previous studies that investigated sex differences in physical activities in adolescents did not focus on weekdays and weekends specifically. Moreover, many previous studies focused on comparing the amount of moderate-to-vigorous physical activity [41], while the fPCA derived profiles did not specifically focus on a specific time period or apply a prefixed cutoff to define physical activity intensities. Thus, the high amplitude profile reflects the overall physical activity pattern that include both high- and low-intensity activities. Indeed, a more recent study using NHANES 2011–2014 data to compare total activity levels between boys and girls reported findings similar to ours [42].

### Early activity window profile

PCA2 captured the timing of the active phase and explained 21.1–24.4% of total variance. The timing aspect was also captured in previous fPCA studies in adults, explaining 11.4–23.0% of variance [5–9], supporting the timing aspect as an important feature of the 24-hour rest-activity patterns. The timing of the activity window is directly related to sleep timing, and both are determined by many internal and external factors. Previous studies have consistently established an age-related delay in chronotype (i.e., time preference of the sleep window) during adolescence [43], which is consistent with the later activity window found in the oldest age group in our study. The fact that both weekday and weekend PCA2 were associated with older age suggested this association may be primarily driven by biological rather than environmental factors such as school schedule. Although it is unclear what factors drove this shift in chronotype, some studies suggested it could be partly due to changes in circadian patterns of hormones during adolescence [44–46].

We also observed an earlier active window in Mexican American compared with white participants. Few studies have examined racial and ethnical differences in either activity timing or chronotypes, but the result for Mexican Americans was directionally similar to findings in the previous fPCA study in adults [5]. It is also noteworthy that the relationship was not observed on weekends, suggesting that factors contributing to an earlier activity window in Mexican Americans are likely weekday specific. It would be interesting for future studies to investigate underlying contributors, internal or external, to such racial and ethnic differences. Adolescents with the lowest household income had a later active phase, and a similar association was reported in the previous study in the NHANES adult [5]. However, our study is the first that examined the relationship between family socioeconomic status and activity timing in adolescent, and more evidence is needed to clarify this relationship.

### Early activity peak profile

This profile, which explained slightly less than 10% of total variance, was distinct from the early activity window profile (PCA2) in that instead of featuring a temporal shift of the active phase, it is characterized by the timing of peak activity within the active phase. The only characteristic that was associated with this profile was age, with the oldest age group (18-19.9) exhibiting the highest eigenvalue. Notably, the association between different age groups and the profile characterizing the timing of peak activity differed for weekday (PCA3) and weekends (PCA4), suggesting that this profile may be partially affected by external factors, such as school schedule and after-school sports activities. Specifically, the earlier timing of peak activity among the 18 + age group may be driven by a lower participation in after-school sports training after graduating from high school. Recently, a number of studies suggested that the timing of exercise may have an impact on its health effects [47, 48], however the evidence is still limited and research among adolescents is lacking. Future studies should examine how timing of exercise may affect physical and mental health in adolescents, which will provide valuable information for designing school-based and extracurricular exercise programs among children and adolescents.

### Prolonged activity/reduced rest window profile

The length of day-time activity was captured by the PCA4 which explained 7.1–7.7% variance. A similar profile was reported in previous fPCA studies in adults, explaining 9.1-14.8% variance [5–9]. Since the 24-h rest-activity cycle can be divided into an active and a resting phase, a higher value of this profile therefore indicates both a prolonged window for activity and a reduced window for rest or sleep. Thus, the more prolonged activity window found in the older adolescents essentially reflected a shorter sleep duration, which is consistent with the results found in the Youth Risk Behavior Surveys showing U.S. students in grade 12 had a higher prevalence of short sleep duration than students in grade 9 (77.6% vs. 65.6%) [49]. Another nationally representative study of the U.S. adolescents also found those aged 18–19 were 68–75% less likely to report ≥ 7 h of sleep per night, compared with those aged 12–13 [15]. In addition, girls on average showed a more prolonged activity window than boys did in our analysis, which is consistent with previous studies which showed adolescent girls in the U.S. had a higher prevalence of short sleep duration than boys (75.6% vs. 69.9%) and were about 30% less likely to report ≥ 7 h of sleep per night [15, 49]. A few mechanisms have been proposed to explain shorter sleep duration in girls relative to boys, including hormonal changes during puberty, higher stress and mental problems [50–52]. However, such sex difference was only observed for weekdays, not weekends, suggesting social factors may play an important role.

Non-Hispanic Black participants on average showed a higher eigenvalue for this profile than Non-Hispanic White participants, which is consistent with findings in NHANES adults [5]. This association may be primarily driven by a shorter sleep duration in the Black participants. Multiple studies reported a shorter sleep duration in Black adolescents than White adolescents [15, 49, 53]. Such racial disparities in sleep may be explained by various factors, including environmental disturbances such as light and noise due to poor housing and neighborhood conditions [54, 55], higher stress [56], and a higher prevalence of chronic conditions such as obesity and cardiovascular diseases in African Americans [53, 57]. Similar to sex differences, racial difference in this profile appeared stronger on weekdays, while the Black-White difference almost vanished on weekends. This suggested that environmental constrains specific to weekdays (e.g., school schedules, commute, school work) may have contributed the disparities in sleep duration between racial groups.

### Strengths and limitations

A major strength of our study is the use of fPCA to derive activity profiles that are important for explaining population-level differences in 24-hour rest-activity patterns. Compared to other commonly used methods for characterizing rest and activity behaviors, including measurements of total physical activity levels and moderate-to-vigorous physical activities, duration of sedentary behaviors and/or sleep, and metrics derived from cosine-based models or non-parametric algorithms (e.g., interdaily stability, intradaily variability), a notable strength of fPCA is its flexible, data-driven, and shape-naïve approach. Specifically, fPCA does not rely on prefixed intensity cutoffs or assumptions about 24-hour shapes of rest-activity rhythms, which are often insensitive to differences in activity levels and patterns that vary according to population characteristics (e.g., sociodemographic compositions, health status). As a result, the fPCA is able to capture the most prominent activity profiles or features specific to a study sample. This is a particular strength for our study, which utilized a representative sample of American adolescents, because profiles derived from our analysis are reflective of rest-activity patterns on a national scale and thus have important public health implications. Moreover, the data-driven and flexible approach of fPCA has allowed us to uncover profiles that represent not only previously established crucial domains of rest and activity patterns (e.g., amplitude), but also novel features that have not been documented by earlier studies: For example, we found that the early peak profile was a unique feature distinct from the early activity window profile, which revealed a potentially important nuance in the timing of rest-activity rhythms that has not been reported before.

Beside the application of fPCA, our study has several additional strengths. The data from NHANES allowed us to assess the rest-activity profiles in a national representative sample of U.S. adolescents. The large sample size made it possible to compare across sociodemographic subgroups. The use of accelerometer data provided objective and accurate rest-activity measurements. We also stratified the analysis by weekdays and weekends to examine any differences between schooldays and weekends, which provided important evidence about the role of social and environmental factors in shaping rest-activity patterns in this population.

Despite the strengths, our study also has limitations. First, the rest-activity profiles derived by fPCA are sample specific which tend to weaken external validity. However, we believe the use of the national representative sample in NHANES allows our findings to be generalized to the wide population of American adolescents. However, more studies are warranted to investigate special populations, particularly those with underlying health conditions that affect the rest-activity rhythm, such as sleep and mental disorders. Second, the data were collected from 2011 to 2014, therefore, the activity patterns and the associations observed in our study may not necessarily apply to adolescents in different birth cohorts and/or periods. Third, the excluded participants were generally older and more likely from the highest household income group, compared with the included participants. Therefore, the external validity may be reduced and the results should be interpreted with caution. Fourth, the profiles generated from fPCA could sometimes be difficult to interpret. However, the rest-activity profiles described in our study provided distinct and interpretable features which captured characteristics that were also observed in previous fPCA studies. Fifth, the accelerometer data in NHANES only captured the levels of activity and do not provide information about the specific types of activity or in what context the participants were engaging in these activities. Additionally, since the accelerometer was worn on the wrist, it may not accurately measure activities that primarily involve the lower body. Future studies are needed to examine the specific types of activities in which adolescents participate, and explore their association with demographic characteristics and health outcomes. Lastly, we only examined five demographic and socioeconomic status characteristics. Other factors, such as lifestyle and environmental factors, may also impact the rest-activity profiles and need to be examined by future studies.

## Conclusions

Using a nationally representative sample of adolescents in the U.S., our study identified the four main rest-activity profiles, suggesting the amplitude, timing of activity, and length of sleep/rest are important domains of rest and activity behaviors in adolescents, providing tangible targets for public health monitoring and interventions. Additionally, our results also showed sociodemographic characteristics such as age and race/ethnicity may shape the adolescents’ rest and activity behaviors. Future studies could further validate the associations and investigate interventions targeting vulnerable subgroups of adolescents to enhance their rest-activity rhythms.

### Electronic supplementary material

Below is the link to the electronic supplementary material.

**Supplementary Material 1**: Supplementary figures and tables.

## Data Availability

All NHANES datasets used in the current analysis are available for download from the NHANES website: www.cdc.gov/nchs/nhanes/.

## List of Abbreviations

NHANES: National Health and Nutrition Examination Survey
fPCA: Functional principal component analysis
MIMS: Monitor Independent Movement Summary
