# Supplementary material for: U.S. Adolescent Rest-Activity patterns: insights from functional principal component analysis (NHANES 2011–2014)
Source: Int J Behav Nutr Phys Act. 2023 Oct 13;20:125. doi: 10.1186/s12966-023-01520-3 (PMC10571346; doi:10.1186/s12966-023-01520-3)
Supplement: Supplementary file 1 — Supplementary Material 1: Supplementary figures and tables. [file 12966_2023_1520_MOESM1_ESM.docx]

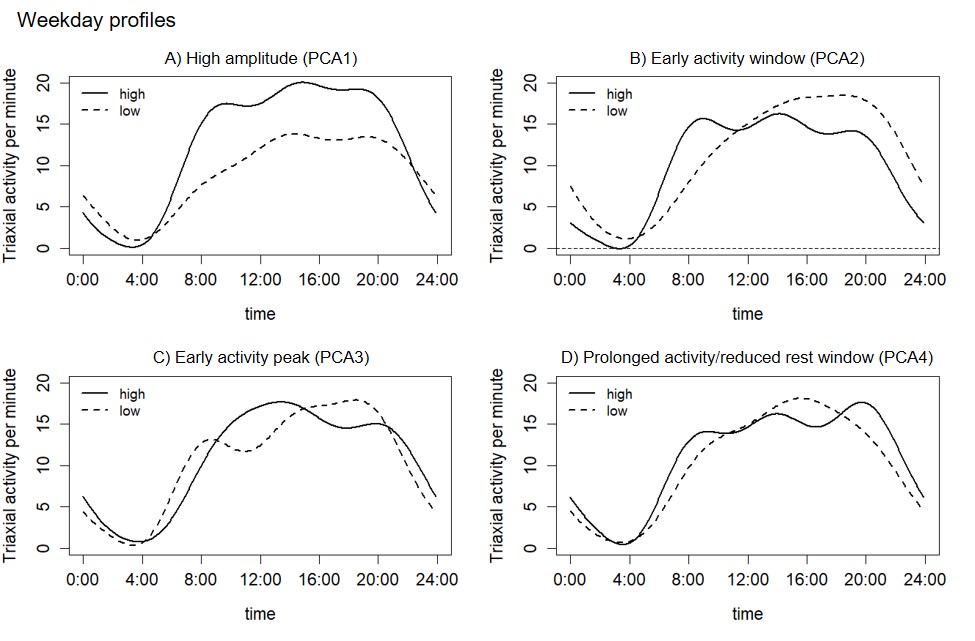


Figure S1 Rest-activity profiles of weekdays 24-hour actigraphy data from adolescents in the National Health and Nutrition Examination Survey (2011-2014). Each panel depicts the mean 24-hour activity patterns for participants with high (solid line) and low (dotted) eigenvalues of the first four components derived from the functional principal component analysis (PCA): A The first component (39.0% variance), with higher eigenvalues representing a higher amplitude; B the second component (23.8% variance), with higher eigenvalues representing earlier activity window; C the third component (9.9% variance), with higher eigenvalues representing earlier peak of daytime activity; D the forth component (7.7% variance), with higher eigenvalues representing prolonged activity/reduced rest window


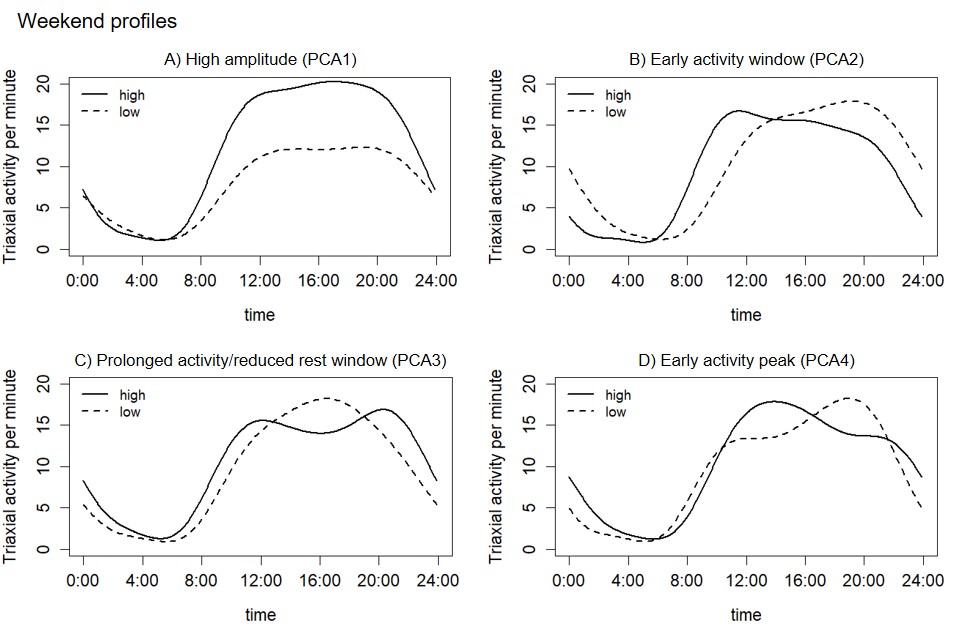


Figure S2 Rest-activity profiles of weekends 24-hour actigraphy data from adolescents in the National Health and Nutrition Examination Survey (2011-2014). Each panel depicts the mean 24-hour activity patterns for participants with high (solid line) and low (dotted) eigenvalues of the first four components derived from the functional principal component analysis (PCA): A The first component (39.4% variance), with higher eigenvalues representing a higher amplitude; B the second component (21.1% variance), with higher eigenvalues representing earlier activity window; C the third component (9.8% variance), with higher eigenvalues representing a more prolonged activity/reduced rest window; D the forth component (8.9% variance), with higher eigenvalues representing earlier peak of daytime activity


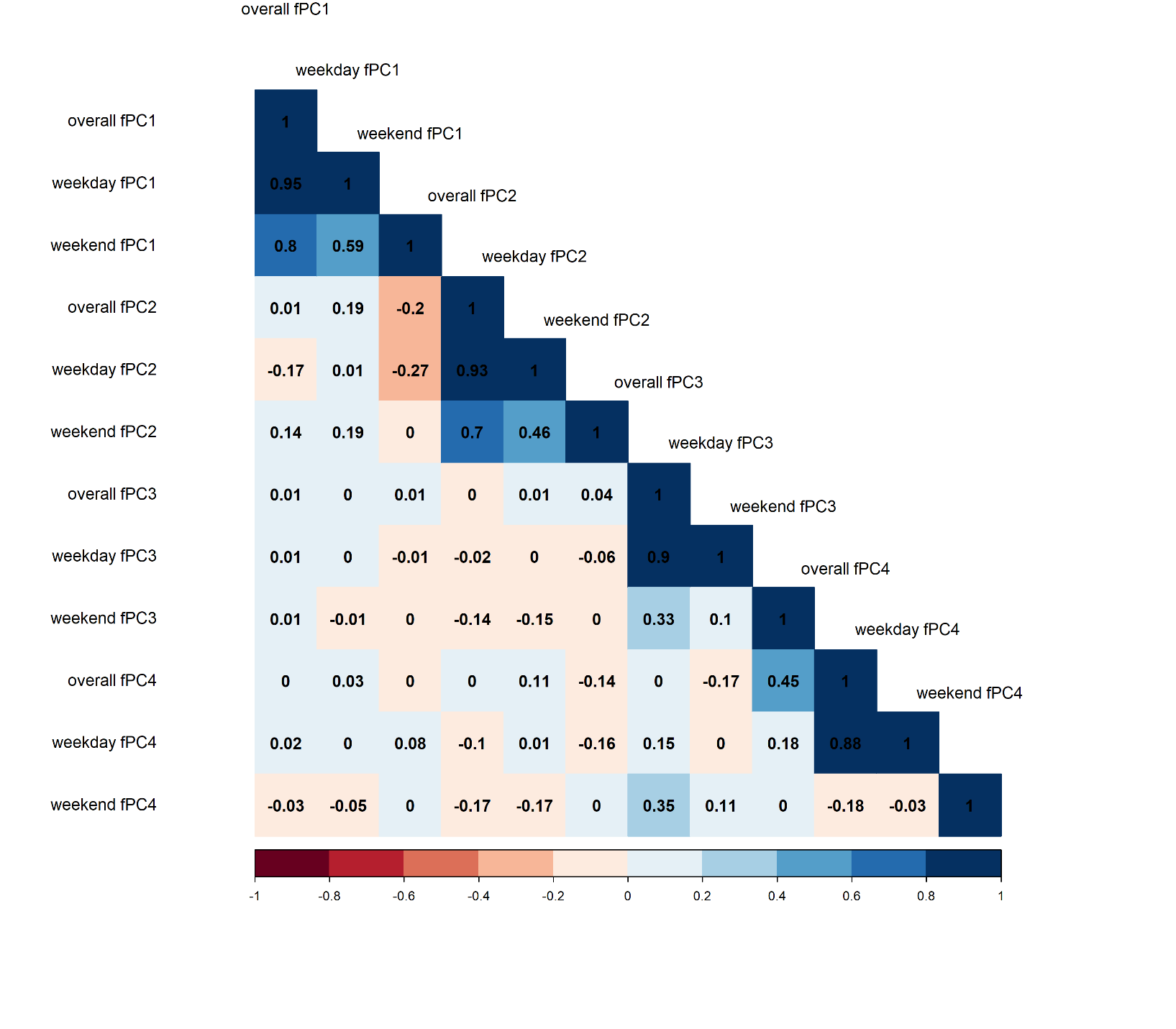

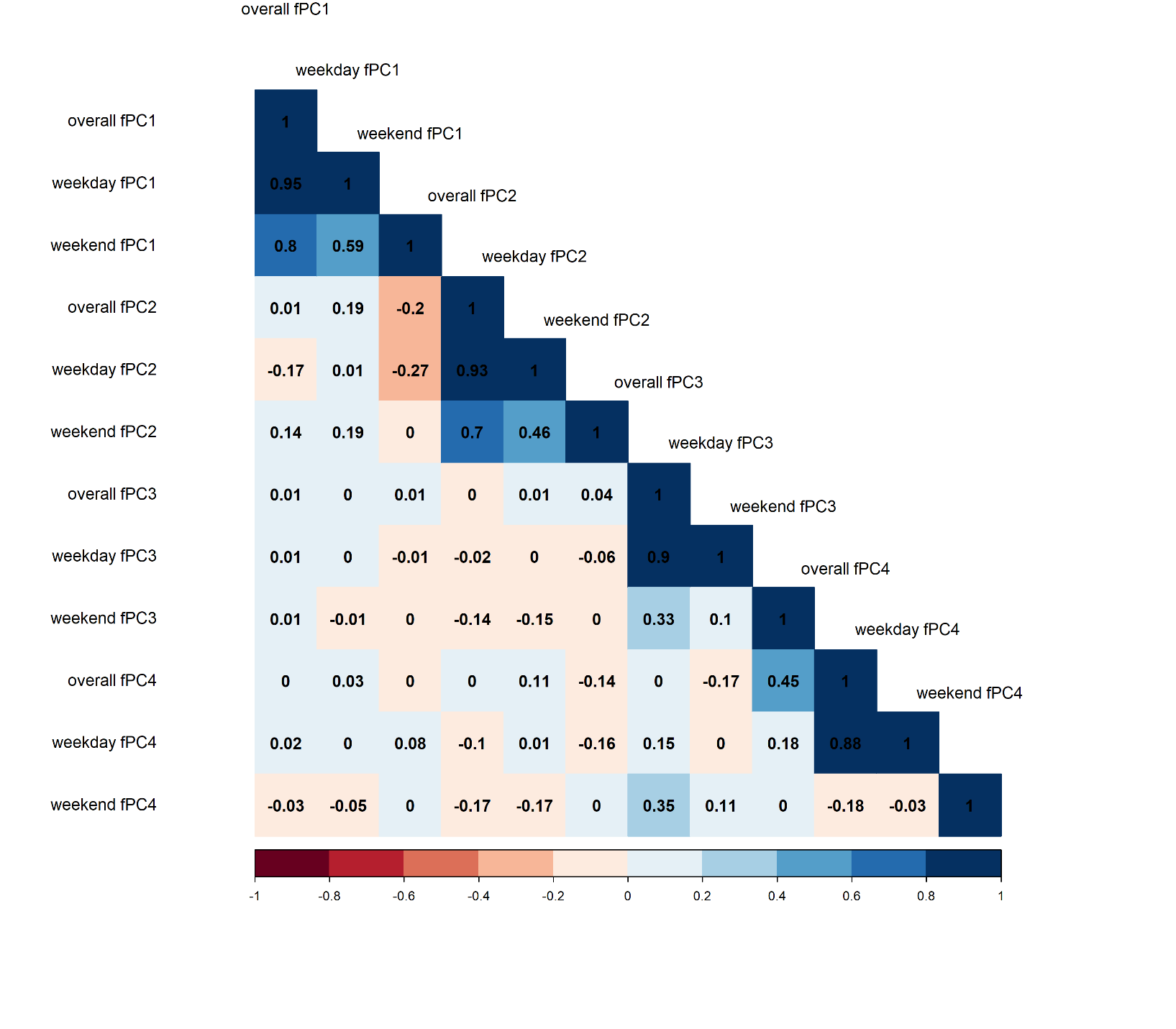


Figure S3 Correlation matrix plot between overall, weekday and weekend rest-activity profiles among adolescents in the National Health and Nutrition Examination Survey (2011-2014).


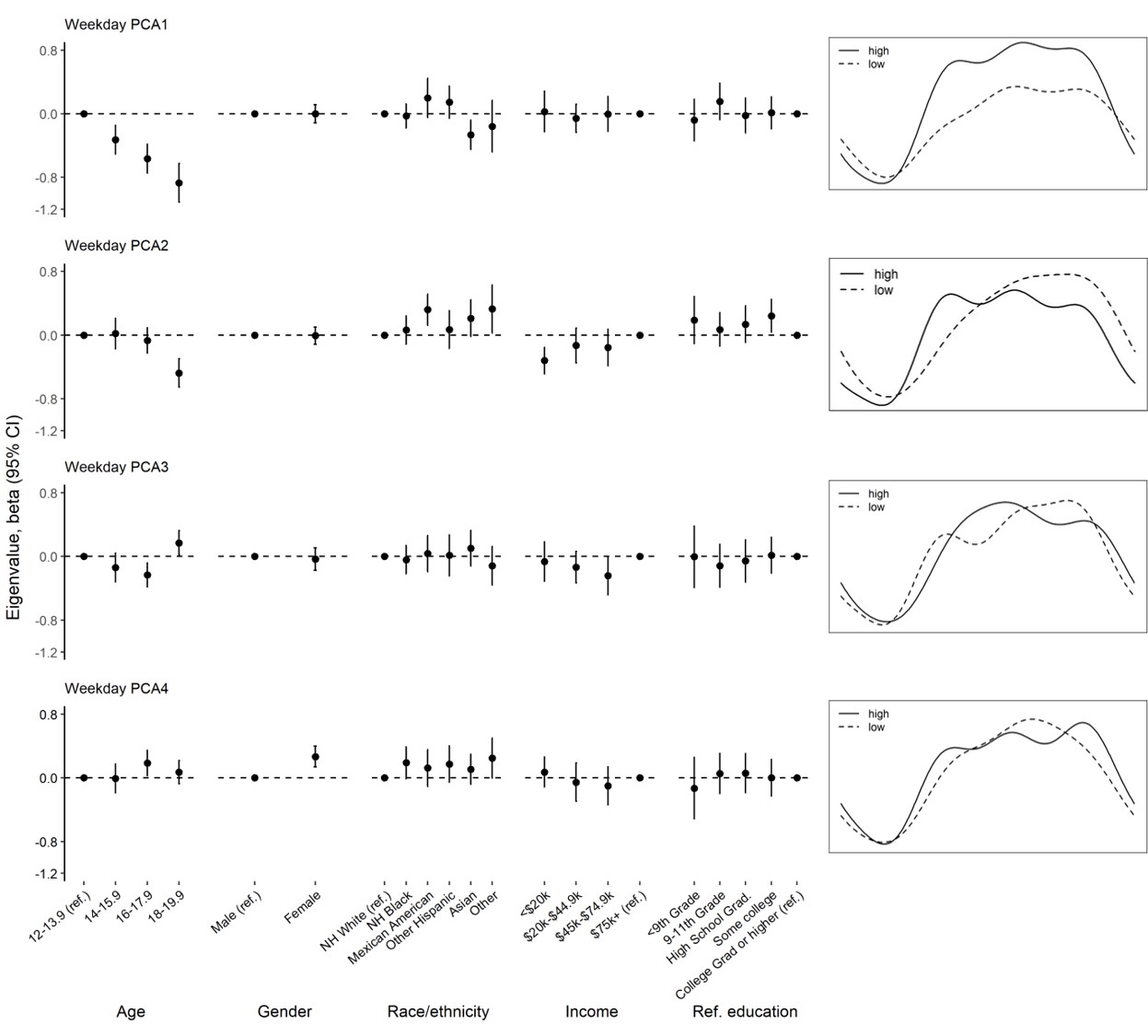


Figure S4 Associations between participant characteristics and weekday rest-activity profiles in adolescence in the National Health and Nutrition Examination Survey (2011-2014). Multiple linear regression models included all participant characteristics simultaneously. Abbreviations: CI, confidence interval; HS, high school; NH, non-Hispanic; PCA1-4, principal component analysis component 1-4


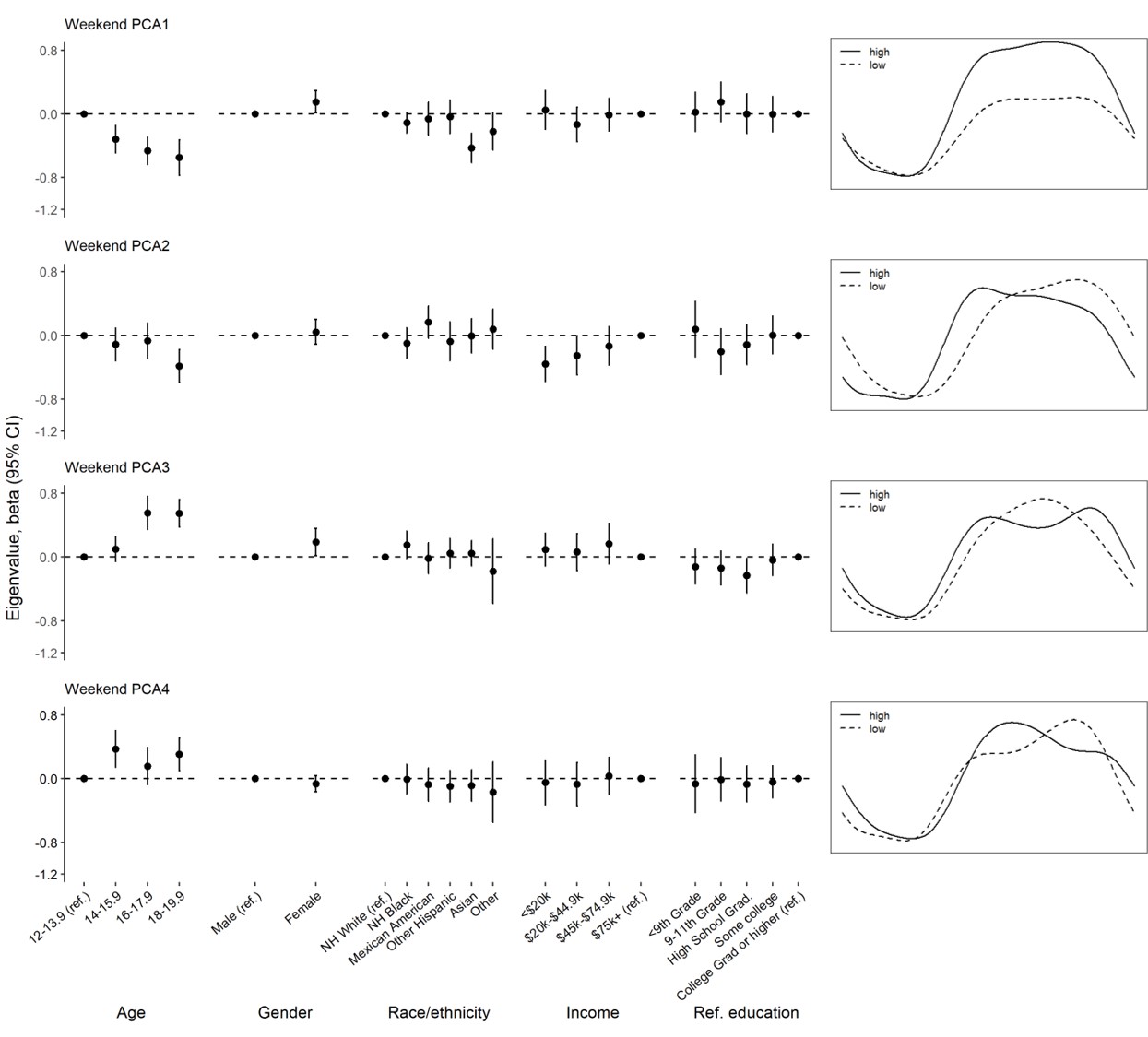


Figure S5 Associations between participant characteristics and weekend rest-activity profiles in adolescence in the National Health and Nutrition Examination Survey (2011-2014). Multiple linear regression models included all participant characteristics simultaneously. Abbreviations: CI, confidence interval; HS, high school; NH, non-Hispanic; PCA1-4, principal component analysis component 1-4

Table S1 Comparisons between included and excluded participants who aged 12-19, National Health and Nutrition Examination Survey (2011-2014)

|  | **Excluded** | **Included** | **P-value^a^** |
| --- | --- | --- | --- |
| N (%) | 891 (33) | 1841 (67) |  |
| Age, % |  |  | <0.001 |
| 12-13.9 | 18 | 29 |  |
| 14-15.9 | 31 | 24 |  |
| 16-17.9 | 24 | 24 |  |
| 18-19.9 | 27 | 23 |  |
| Sex, % |  |  | 0.1 |
| Male | 55 | 49 |  |
| Female | 45 | 51 |  |
| Race/ethnicity, % |  |  | 0.8 |
| NH Black | 15 | 15 |  |
| Mexican American | 14 | 15 |  |
| Other Hispanic | 6.7 | 7.4 |  |
| Asian | 4.4 | 5 |  |
| Other | 4.3 | 4.1 |  |
| NH White | 56 | 54 |  |
| Household income, % |  |  | 0.03 |
| <20k | 14 | 15 |  |
| 20-44.9k | 25 | 27 |  |
| 45-74.9k | 15 | 19 |  |
| >75k | 37 | 33 |  |
| HH ref person's education level, % |  |  | 0.2 |
| Less Than 9th Grade | 5.4 | 6.8 |  |
| 9-11th Grade | 11 | 10 |  |
| High School Grad | 21 | 17 |  |
| Some College | 32 | 31 |  |
| College Graduate | 30 | 33 |  |

Values were weighted using sample weights, other than sample size (N).
**^a^** P-values were derived from Chi-square test.
Abbreviations: HH Household, NH non-Hispanic

Table S2 Pearson correlation coefficients between overall, weekday and weekend rest-activity profiles among adolescents in the National Health and Nutrition Examination Survey (2011-2014).

|  |  | **PCA1** | | | **PCA2** | | | **PCA3** | | | **PCA4** | | |
| --- | --- | --- | --- | --- | --- | --- | --- | --- | --- | --- | --- | --- | --- |
|  |  | **Overall** | **Weekday** | **Weekend** | **Overall** | **Weekday** | **Weekend** | **Overall** | **Weekday** | **Weekend** | **Overall** | **Weekday** | **Weekend** |
| **PCA1** | **Overall** | 1.00 | 0.95 | 0.80 | 0.01 | -0.17 | 0.14 | 0.01 | 0.01 | 0.01 | 0.00 | 0.02 | -0.03 |
|  | **Weekday** |  | 1.00 | 0.59 | 0.19 | 0.01 | 0.19 | 0.00 | 0.00 | -0.01 | 0.03 | 0.00 | -0.05 |
|  | **Weekend** |  |  | 1.00 | -0.20 | -0.27 | 0.00 | 0.01 | -0.01 | 0.00 | 0.00 | 0.08 | 0.00 |
| **PCA2** | **Overall** |  |  |  | 1.00 | 0.93 | 0.70 | 0.00 | -0.02 | -0.14 | 0.00 | -0.10 | -0.17 |
|  | **Weekday** |  |  |  |  | 1.00 | 0.46 | 0.01 | 0.00 | -0.15 | 0.11 | 0.01 | -0.17 |
|  | **Weekend** |  |  |  |  |  | 1.00 | 0.04 | -0.06 | 0.00 | -0.14 | -0.16 | 0.00 |
| **PCA3** | **Overall** |  |  |  |  |  |  | 1.00 | 0.90 | 0.33 | 0.00 | 0.15 | 0.35 |
|  | **Weekday** |  |  |  |  |  |  |  | 1.00 | 0.10 | -0.17 | 0.00 | 0.11 |
|  | **Weekend** |  |  |  |  |  |  |  |  | 1.00 | 0.45 | 0.18 | 0.00 |
| **PCA4** | **Overall** |  |  |  |  |  |  |  |  |  | 1.00 | 0.88 | -0.18 |
|  | **Weekday** |  |  |  |  |  |  |  |  |  |  | 1.00 | -0.03 |
|  | **Weekend** |  |  |  |  |  |  |  |  |  |  |  | 1.00 |
